# MicroRNA-608 inhibits proliferation of bladder cancer via AKT/FOXO3a signaling pathway

**DOI:** 10.1186/s12943-017-0664-1

**Published:** 2017-05-26

**Authors:** Zhen Liang, Xiao Wang, Xin Xu, Bo Xie, Alin Ji, Shuai Meng, Shiqi Li, Yi Zhu, Jian Wu, Zhenghui Hu, Yiwei Lin, Xiangyi Zheng, Liping Xie, Ben Liu

**Affiliations:** 1 Department of Urology, the First Affiliated Hospital, Zhejiang University, School of Medicine, 79, Qingchun Road, 310003 Hangzhou, Zhejiang China; 2 Department of Urology, TongDe Hospital of Zhejiang Province, Hangzhou, China; 3 Department of Urology, Zhejiang Provincial People’s Hospital, Hangzhou, China

**Keywords:** Bladder cancer, Proliferation, MicroRNA-608, FLOT1

## Abstract

**Background:**

Current evidence indicates that miR-608 is widely down-regulated in various malignant tumors including liver cancer, colon cancer, lung cancer and glioma, and acts as a tumor suppressor by inhibiting cell proliferation, invasion and migration or by promoting apoptosis. The specific biological function of miR-608 in bladder cancer is still unknown.

**Methods:**

qRT-PCR and Chromogenic in Situ Hybridization (CISH) was conducted to assess the expression of miR-608 in paired BCa tissues and adjacent non-tumor bladder urothelial tissues. Bisulfite sequencing PCR was used for DNA methylation analysis. CCK-8, colony formation and flow cytometry assays were performed, and a xenograft model was studied. Immunohistochemistry staining was performed with peroxidase and DAB. The target of miR-608 was validated with a dual-luciferase reporter assay, quantitative RT-PCR, and Western blotting.

**Results:**

miR-608 is frequently down-regulated in human BCa tissues. The methylation status of CpG islands is involved in the regulation of miR-608 expression. Overexpression of miR-608 inhibits the proliferation and tumorigenesis of BCa cells in vitro and in vivo. Additionally, up-regulation of miR-608 in BCa cells induces G1-phase arrest through AKT/FOXO3a signaling. In contrast, down-regulation of miR-608 promotes proliferation and cell cycle progression in BCa cells. Moreover, the expression of FLOT1 was directly inhibited by miR-608, the down-regulation of FLOT1 induced by siFLOT1 could be significantly reversed by miR-608 inhibitor. Similarly, the up-regulation of FLOT1 by FLOT1 overexpression plasmid (pFLOT1) could also reverse the suppressed cell proliferation caused by miR-608.

**Conclusions:**

miR-608 is a potential tumor suppressor in BCa, and the restoration of miR-608 might be a promising therapeutic option for BCa.

**Electronic supplementary material:**

The online version of this article (doi:10.1186/s12943-017-0664-1) contains supplementary material, which is available to authorized users.

## Background

Bladder cancer (BCa) is the most common malignant tumor of the urogenital tract. It ranks as the 7th most common cancer in men and 17th most common cancer in women [1, 2]. In the United States, BCa ranks as the 4th among all malignant tumors. Approximately 76,960 new BCa cases (58,950 males and 18,010 females) and 16,390 deaths of BCa (11,820 males and 4570 females) were expected in 2016 [3]. Although 75% of newly diagnosed bladder cancers are noninvasive [1], and localized BCa can be managed by surgery, there are one-third of BCa cases still recurring and progressing to locally invasive or metastatic stages [4]. In patients with progressed bladder cancer, the therapeutic prognosis remains unstatisfactory. The lack of effective therapies for the progression of bladder cancer urges for studies on the molecular etiology and identification of novel therapeutic targets including non-coding RNAs.

MicroRNAs (miRNAs) are a family of short (20 ~ 24 nucleotides), endogenous noncoding RNAs which are vital gene regulators binding to partially complementary sequences at the 3′ untranslated regions (3′-UTR) of mRNAs and directing post-transcriptional modulation [5]. It has been reported that more than 1000 miRNAs are encoded by the mammalian genome [6], and all the miRNAs are estimated to target over 5300 human genes representing 30% of the human gene set [7]. The dysregulation of miRNAs has been discovered in multiple cancers, and is involved in various physiological and pathological processes of tumor cells, such as proliferation, differentiation, apoptosis, metabolism, metastasis and cell signaling [8–12]. Recent studies have indicated that the abnormal expression of miRNAs also plays an important role in the tumorigenesis and development of bladder cancer [13–16]. Our previous studies have identified various miRNAs functioning as tumor suppressors in bladder cancer, including miR-101, miR-124-3p, miR-320c, miR-433, miR-409-3p, miR-490-5p, and miR-576-3p, which regulate the proliferation, migration and invasion of bladder cancer cells by down-regulating various oncogenes [17–23].

MicroRNA-608 is a newly identified miRNA transcribed from the genetic locus located at human chromosome 10q24.31, and this locus also lies in an intron of the SEMA4G gene. Current evidence indicates that miR-608 is widely down-regulated in various malignant tumors including liver cancer, colon cancer, lung cancer and glioma and acts as a tumor suppressor by inhibiting cell proliferation, invasion, migration or by promoting apoptosis [24–27]. The specific biological function of miR-608 in bladder cancer is still unknown. In our research, for the first time, we discovered the widespread down-regulation of miR-608 in human bladder cancer tissues. Furthermore, we revealed that miR-608 could suppress the proliferation and tumorigenesis of bladder cancer cells via targeting FLOT1.

## Results

### miR-608 is down-regulated in BCa

**Fig. 1:**
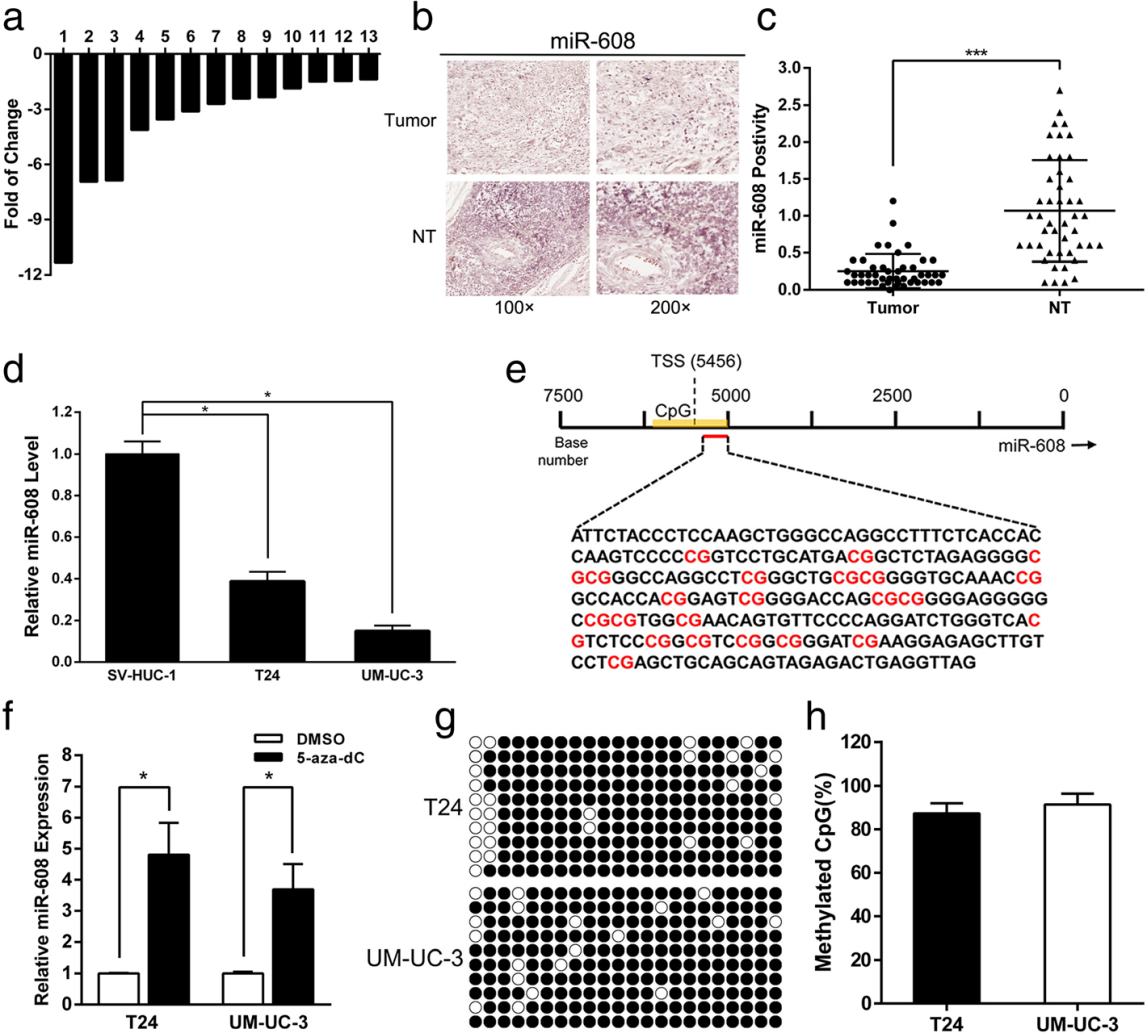
miR-608 is down-regulated in BCa and regulated by DNA methylation. **a** The relative expression levels of miR-608 in 13 pairs of BCa tissues were presented as the fold change of miR-608 referred to the corresponding normal tissues. **b** Representative CISH staining of miR-608 in TMA. **c** Statistical analysis indicated that the expression levels of miR-608 in BCa tissues were significantly higher than those in adjacent non-tumor tissues in TMA. **d** The relative miR-608 levels in BCa cell lines (T24 and UM-UC-3) compared with non-tumor urothelial cell line (SV-HUC-1). **e** The DNA sequence of CpG island in miR-608 promoter analyzed by BSP. **f** In contrast with DMSO, demethylation agent 5-aza-dC stimulated the expression of miR-608 in BCa cell lines (T24 and UM-UC-3). Error bars represent the S.D. from three independent experiments. **g** and **h** Methylation profile in T24 and UM-UC-3 cell lines. The open and filled circles symbolized the unmethylated and methylated CpGs respectively. Ten colonies from each cell line were analyzed. Error bars represent the S.D. from ten randomly chosen colonies. *P < 0.05

In order to verify the expression pattern of miR-608 in BCa, we performed quantitative real-time PCR (qRT-PCR) on 13 cases of clinical BCa tissues paired with adjacent normal urothelial tissues (the clinical profiles of the patients are listed in Additional file 1: Table S1), and in two BCa cell lines (T24 and UM-UC-3) in comparison with SV-HUC-1 cells (a normal transitional epithelial cell line). Moreover,we conducted the Chromogenic in Situ Hybridization (CISH) staining of miR-608 in the bladder cancer tissue microarray (TMA). The results indicated that the expression levels of miR-608 in tumor tissues were dramatically down-regulated compared with paired normal urothelial tissues (Fig. 1a, b and c). Similarly, the expression levels of miR-608 in T24 and UM-UC-3 cells were also markedly low in contrast with SV-HUC-1 cells (Fig. 1d). All these results verified the generally low expression pattern of miR-608 in BCa, and implied that miR-608 is a putative suppressor of bladder tumorigenesis.

### The methylation status of CpG islands is involved in the regulation of miR-608 expression

Previous studies have already revealed that the methylation status of CpG islands might relate with the regulation of various miRNAs expression in cancer cells [21, 28–31]. In our study, we first predicted that the transcript start site (TSS) of miR-608 was possibly located 5456 bp upstream of the miR-608 locus by searching the miRStart database (http://mirstart.mbc.nctu.edu.tw/home.php) [32]. Then, we identified an explicit CpG island located near the TSS by using the CpG island searching program:MethPrimer (http://www.urogene.org/methprimer/) (Fig. 1e). We further treated T24 and UM-UC-3 cells with 5-aza-2′-deoxycytidine (5-aza-dc), a DNA methyltransferase inhibitor, to figure out the underlying relation between the methylation status of CpG islands and miR-608 expression. The results showed that the expression of miR-608 sharply elevated in both two cancer cell lines treated with 5-aza-dc (Fig. 1f). Finally, we carried out bisulfite sequencing PCR (BSP) to evaluate the original methylation status of the identified CpG islands in bladder cancer cells. It turned out that DNA methylation levels were extremely high in the two cancer cell lines (T24 and UM-UC-3) with the percentages of 87.3 and 91.4% respectively (Fig. 1g and h). According to all the evidence mentioned above, we proposed that the high methylation status of CpG islands might result in the inhibition of expression of miR-608 in BCa.

### Overexpression of miR-608 inhibits proliferation and tumorigenesis of BCa cells in vitro *and* in vivo

**Fig. 2:**
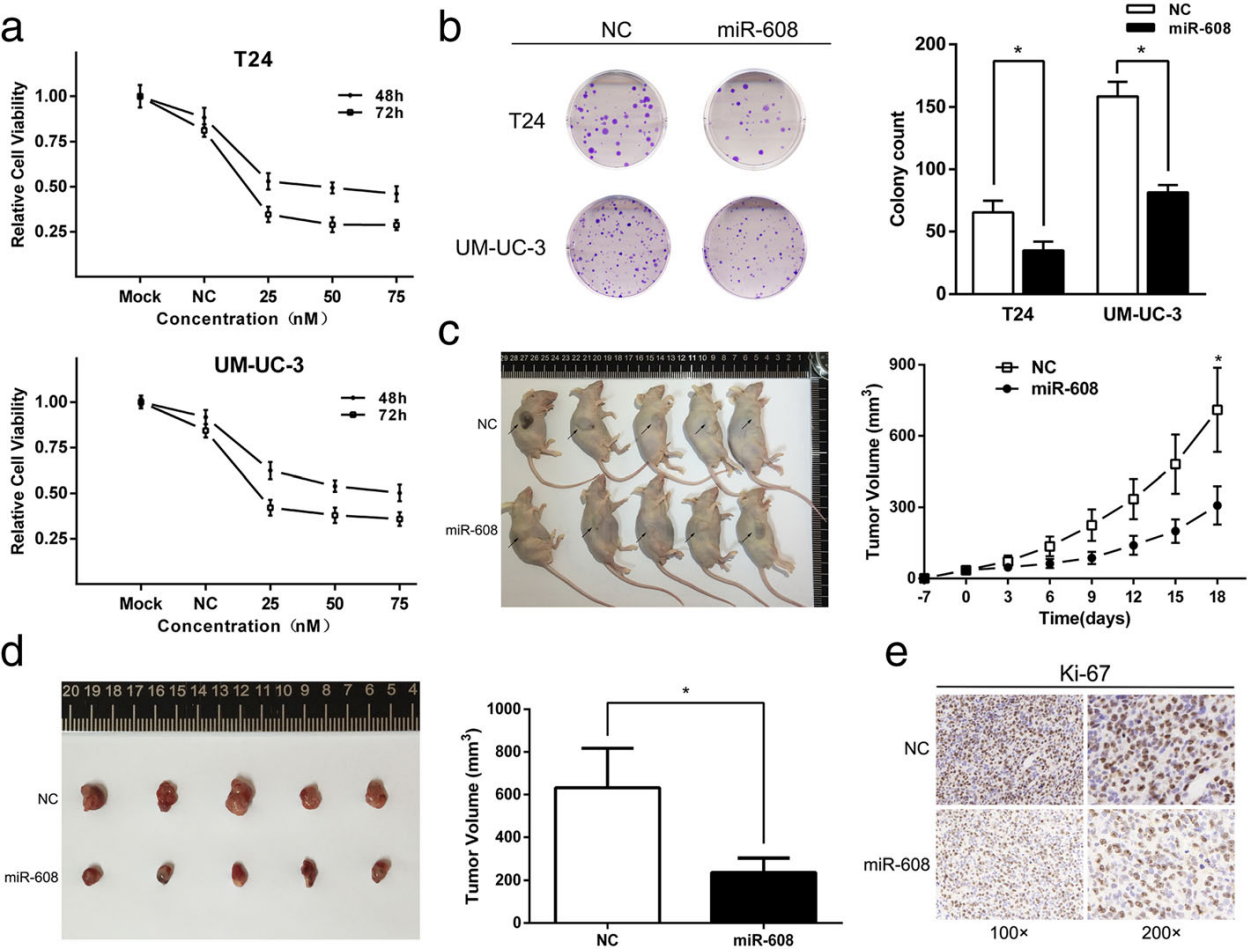
Effect of miR-608 in regulating BCa cell proliferation. **a** CCK-8 assay. The relative viabilities of T24 and UM-UC-3 cells treated with miR-608 were significantly lower than NC treated cells. **b** Colony-formation assay (Representative wells were presented). The colony formation rates of miR-608 transfected BCa cells were obviously lower in contrast with NC transfected cells. Error bars represent the S.D. from three independent experiments. **c**, **d** and **e** Tumor xenograft model. The tumor volumes and the growth curves implied that the growth of tumors in miR-608 group was significantly slower than NC group. Lower Ki-67 expression was also detected in miR-608 treated tumors. Error bars represent the S.D. from five nude mice. *P < 0.05

We transfected T24 and UM-UC-3 cells with miR-608 mimic to figure out the actual effects of miR-608 on cell proliferation. The levels of miR-608 in BCa cells after the transfection of miR-608 mimic were quantified by qRT-PCR (Additional file 2: Figure S1). From the results of CCK-8 assays, we could not detect any obvious dosage effect of miR-608 and miR-608 suppressed the growth of cultured BCa cells at different concentrations and time points (Fig. 2a). We also found out that the overexpression of miR-608 remarkably inhibited the colony formation ability of BCa cells. The colony formation rates of BCa cells after the overexpression of miR-608 were obviously low in contrast with cells transfected with NC (Fig. 2b). After subcutaneous implantation of UM-UC-3 cells into BALB/c mice, we further evaluated the growth rates of BCa cells after overexpression of miR-608 versus NC. It showed that the overexpression of miR-608 could dramatically slow down the growth of tumors in vivo (Fig. 2c and d). In addition, the IHC staining also showed that the Ki-67 indexes of tumors in the miR-608 overexpressed group were lower than those in the control group (Fig. 2e). All these results supported that miR-608 could suppress the growth of BCa cells in vitro *and* in vivo*.*

### Up-regulation of miR-608 in BCa cells induces G1-phase arrest through AKT/FOXO3a signaling

**Fig. 3:**
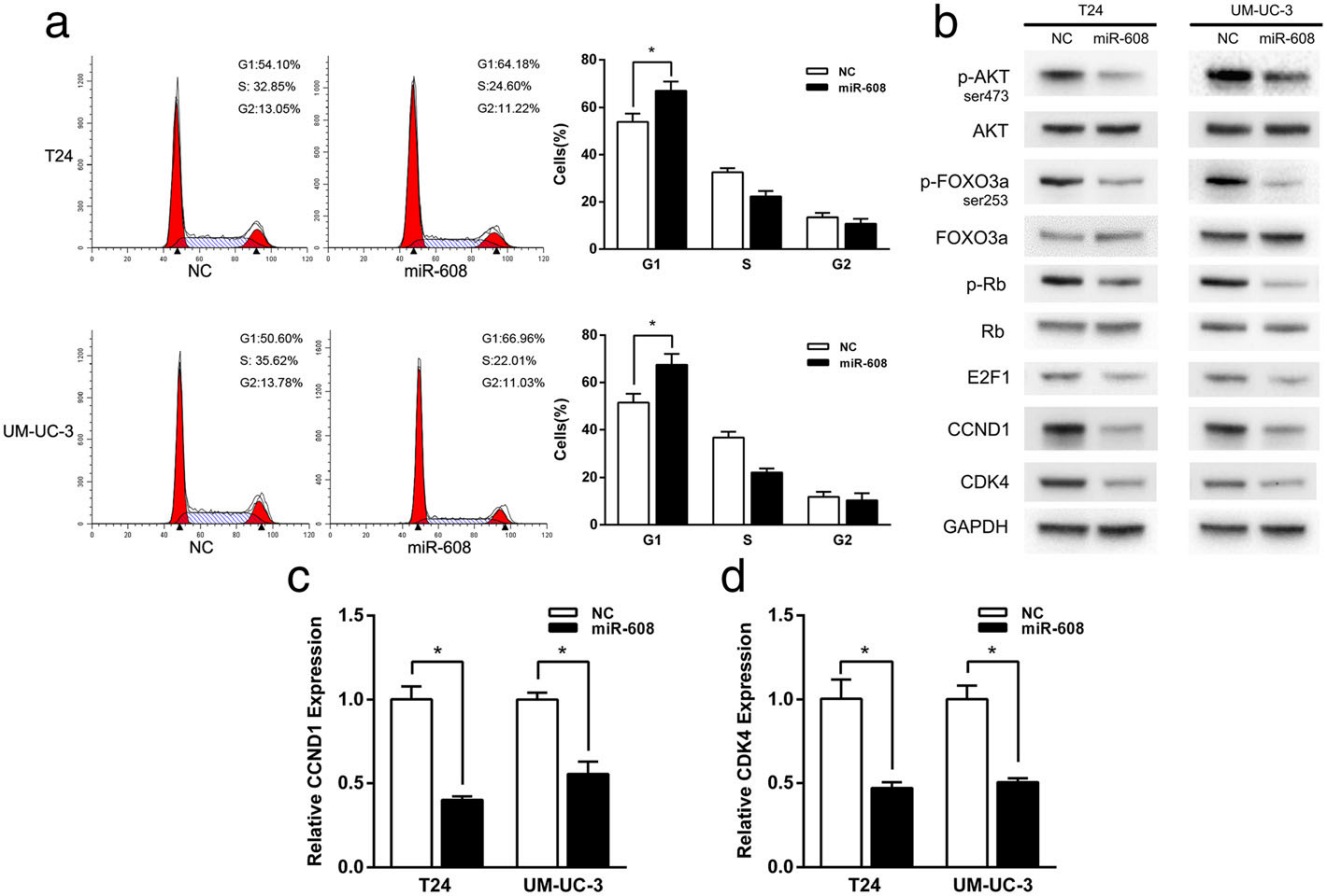
Overexpression of miR-608 inhibits the G1/S transition and cell cycle progression in Bca cells through AKT/FOXO3a signaling. **a** Flow cytometric analysis of cell cycle distribution. Overexpression of miR-608 resulted in a significant G1 phase arrest. **b** Western blot analysis. miR-608 down-regulated AKT/FOXO3a signaling related proteins in T24 and UM-UC-3 cell lines. **c** and **d** qRT-PCR analysis of the expression of CCND1 and CDK4 mRNA in BCa cells (T24 and UM-UC-3) after the transfection of miR-608. Error bars represent the S.D. from three independent experiments. *P < 0.05

We analyzed the possible mechanism of the growth suppression induced by miR-608 in BCa cells by using FACS. It showed that the overexpression of miR-608 in T24 and UM-UC-3 cells significantly increased the percentages of cells in G0/G1 phase and reversely decreased the percentages of cells in S phase (Fig. 3a). At the same time, as the major G1/S transition regulators, the protein and mRNA levels of CCND1 and CDK4 were significantly decreased in miR-608 overexpressed BCa cells, which was consistent with the G1 phase arrest appearance (Fig. 3b, c and d). Furthermore, as the important downstream effectors of cell cycle signaling, the expression levels of p-Rb and E2F1 were also down-regulated (Fig. 3b). It has been reported that FOXO3a is a crucial transcription factor regulating the expression of CCND1 [33, 34], and the transcriptional activity of FOXO3a is inactivated by the phosphorylation catalyzed by p-AKT [35–37]. We then used an AKT inhibitor (LY294002) to verify whether the inhibition of AKT phosphorylation could induce the activation of FOXO3a in BCa cells. The results indicated that the activity of FOXO3a could be dramatically increased by LY294002 in both two BCa cell lines (Additional file 3: Figure S2). According to all these evidence, we suggest that the up-regulation of miR-608 might block the cell cycle progressing through G1-phase in BCa cells by inhibiting AKT/FOXO3a signaling. As manifested in Fig. 3b, levels of p-AKT and p-FOXO3a were both down-regulated in the miR-608 overexpressed BCa cells.

### Down-regulation of miR-608 promotes the proliferation and the cell cycle progression in BCa cells

**Fig. 4:**
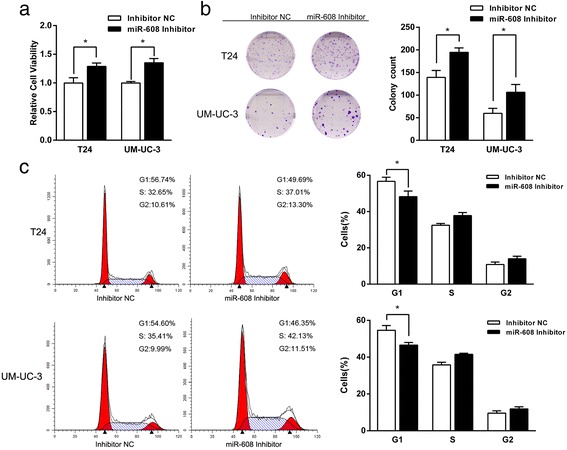
Down-regulation of miR-608 promotes the proliferation and the cell cycle progression in BCa cells. **a** CCK-8 assay. The relative viabilities of T24 and UM-UC-3 cells transfected with miR-608 Inhibitor were significantly higher than Inhibitor NC transfected cells. **b** Colony-formation assay (Representative wells were presented). The colony formation rates of BCa cells treated with miR-608 Inhibitor were higher compared with those treated with Inhibitor NC. **c** Flow cytometric analysis of cell cycle distribution. miR-608 Inhibitor promoted the progression of cell cycle from G1 to S phase in BCa cells. Error bars represent the S.D. from three independent experiments. *P < 0.05

We used the inhibitor of miR-608 to simulate the down-regulation of miR-608 in T24 and UM-UC-3 cells. The levels of miR-608 after the transfection of inhibitor were measured by qRT-PCR (Additional file 4: Figure S3). The outcomes of CCK8 and colony formation assays showed that the down-regulation of miR-608 could significantly increase the proliferation in both of BCa cells (Fig. 4a and b). In addition, contrary to the effect of miR-608 up-regulation, the down-regulation of miR-608 effectively promoted the progression of cell cycle in BCa cells with the decreased percentages of cells in G0/G1 phase and the increased percentages in S phase (Fig. 4c). All the outcomes implied that the down-regulation of miR-608 could accelerated the growth of BCa cells by regulating cell cycle progression.

### miR-608 regulates the expression of FLOT1 by directly targeting the 3′-UTR

**Fig. 5:**
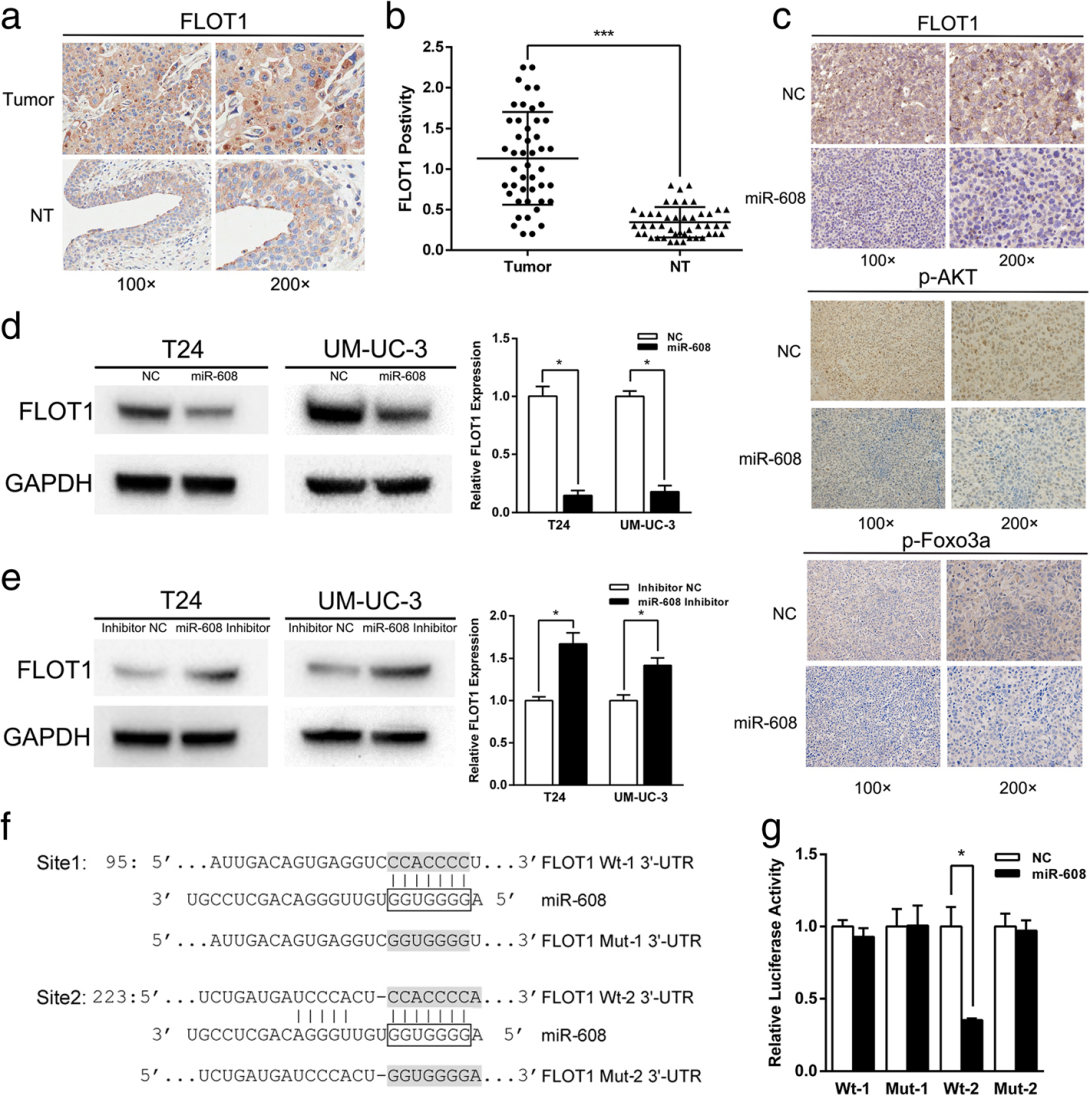
FLOT1 is a direct target of miR-608. **a** Representative IHC staining of FLOT1 in TMA. FLOT1 showed a membranous location. **b** Statistical analysis indicated that the expression levels of FLOT1 protein in BCa tissues were significantly higher than those in adjacent non-tumor tissues in TMA. **c** Representative IHC staining of FLOT1, p-AKT and p-FOXO3a in xenograft tumors treated by miR-608 in contrast with NC. **d** and **e** Western blot and qRT-PCR assays. The protein and mRNA levels of FLOT1 in BCa cells were negatively regulated after the up or down-regulation of miR-608 by using miR-608 mimic or inhibitor respectively. **f** Predicted miR-608 target sequences in the 3′-UTR of FLOT1. **g** Dual-luciferase reporter assay. The relative luciferase activity in HEK 293 T cells co-transfected with miR-608 mimic and Wt-2 vector was sharply inhibited, while in those cells simultaneously transfected with miR-608 mimic and Wt-1 vector or mutant vector (Mut-1 or Mut-2) the luciferase activity was unaffected. Error bars represent the S.D. from three independent experiments. *P < 0.05

Following the basic mechanism of miRNAs in regulating mRNA expression, we attempted to find out the exact targets of miR-608 that were responsible for the inhibition of proliferating by miR-608. By using an online bioinformatics database (TargetScan, http://www.targetscan.org/), we identified FLOT1 as a target of miR-608. There were two high scoring binding sites of miR-608 on the 3′-UTR of FLOT1 mRNA. It has been reported that FLOT1 was the upstream regulator of AKT/FOXO3a pathway in renal cell cancer and the proliferation regulator in BCa and breast cancer [31, 38, 39]. In our study, we discovered that FLOT1 was widely over-expressed in bladder cancer TMAs (Fig. 5a and b), and was significantly down-regulated and accompanied with the decreased p-AKT and p-FOXO3a in miR-608 treated xenograft tumor (Fig. 5c). We also confirmed that both the mRNA and protein levels of FLOT1 in T24 and UM-UC-3 cells were negatively regulated after the up or down-regulation of miR-608 by qRT-PCR and western-blot assays (Fig. 5d and e).

For the purpose of verifying the 3′-UTR of FLOT1 as a direct target of miR-608, the fragment of FLOT1 3′-UTR with either of the predicted wild type miR-608 binding sites (Wt-1 and Wt-2) was separately inserted into the downstream of the firefly luciferase in the pmirGLO Dual-Luciferase miRNA Target Expression Vector, and vector with either of the corresponding mutated binding sites (Mut-1 and Mut-2) were also constructed (Fig. 5f). The relative luciferase activity in HEK 293 T cells co-transfected with miR-608 mimic and Wt-2 vector was sharply inhibited, while in those cells simultaneously transfected with miR-608 mimic and Wt-1 vector or mutant vector (Mut-1 or Mut-2) the luciferase activity was unaffected (Fig. 5g). The results supported that miR-608 could directly inhibit the expression of FLOT1 by binding to the 3′-UTR of its mRNA.

### Suppression of FLOT1 plays a crucial role in miR-608 induced growth inhibition of BCa cells

**Fig. 6:**
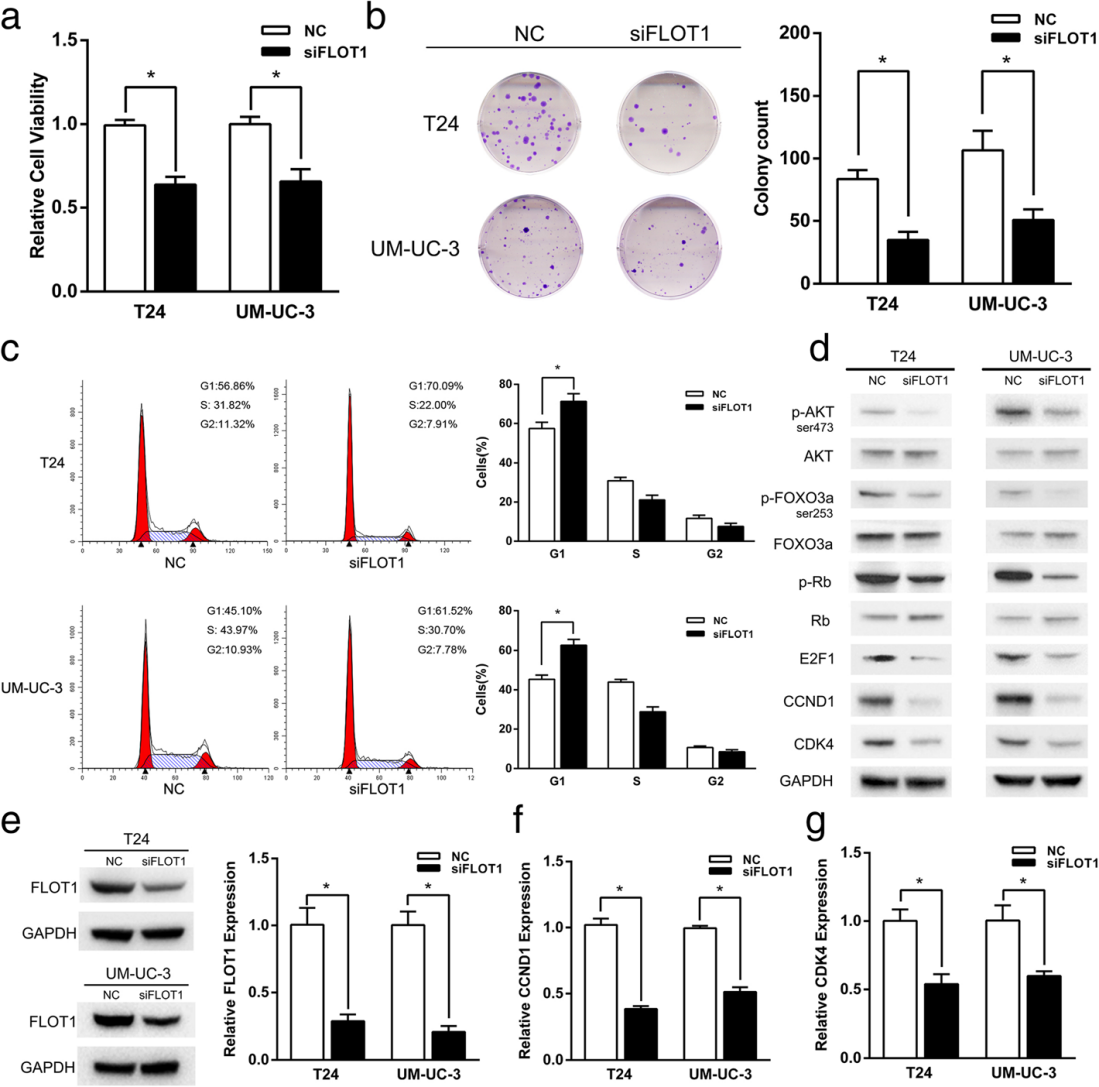
Down-regulation of FLOT1 phenocopied the effect of miR-608. **a** CCK-8 assay. The relative viabilities of T24 and UM-UC-3 cells transfected with siFLOT1 were significantly lower than NC treated cells. **b** Colony-formation assay (Representative wells were presented). The colony formation rates of siFLOT1 transfected BCa cells were sharply lower in contrast with NC transfected cells. **c** Flow cytometric analysis of cell cycle distribution. Knock-down of FLOT1 induced the similar G1 arrest as miR-608 did in BCa cells. **d** Western blot analysis. siFLOT1 down-regulated AKT/FOXO3a signaling related proteins in T24 and UM-UC-3 cell lines. **e** The protein and mRNA levels of FLOT1 were significantly down-regulated by the transfection of siFLOT1. **f** and **g** qRT-PCR analysis of the expression of CCND1 and CDK4 mRNA in BCa cells after the transfection of siFLOT1. Error bars represent the S.D. from three independent experiments. *P < 0.05

**Fig. 7:**
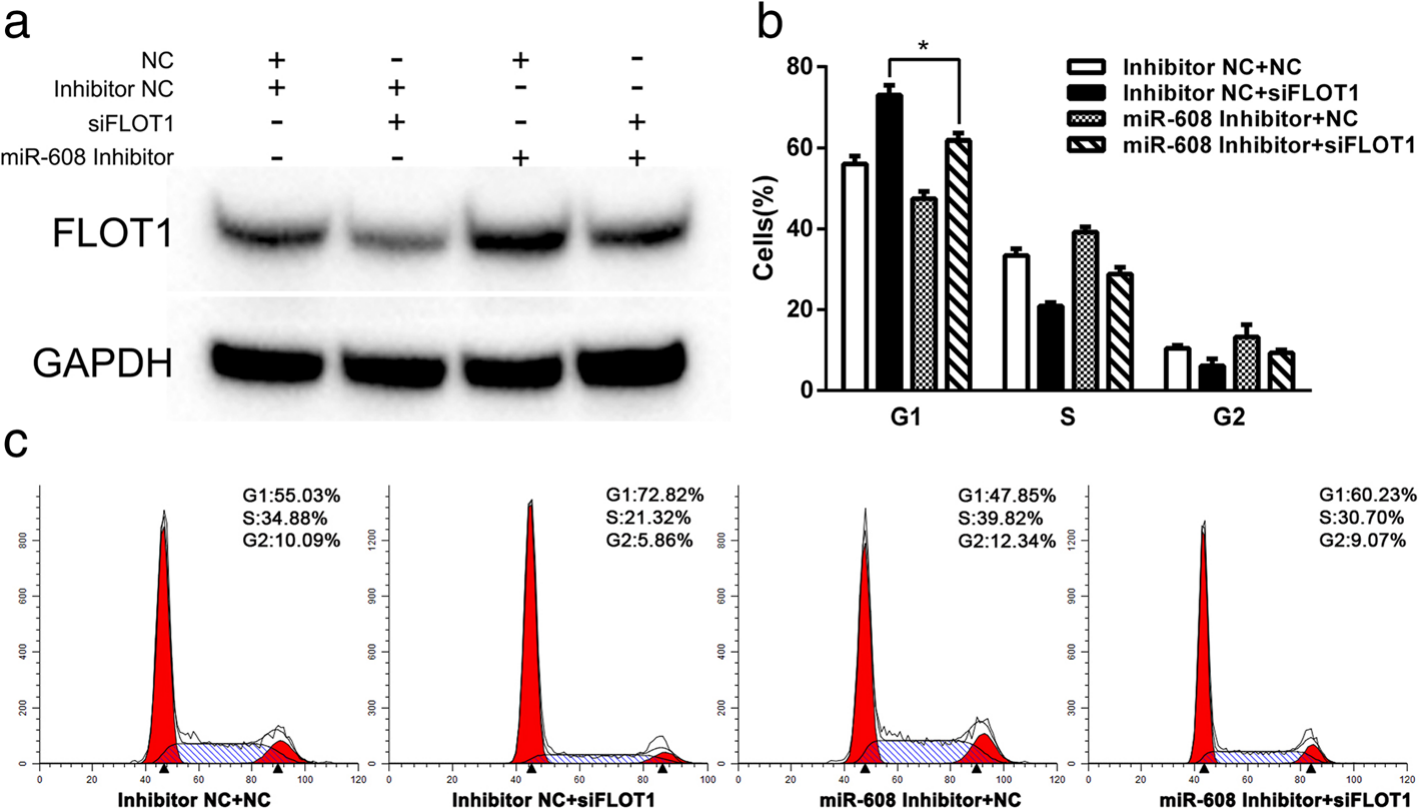
Down-regulation of miR-608 could partially reverse the G1-phase arrest caused by siFLOT1. **a** Western blot analysis. UM-UC-3 cells were co-transfected with miR-608 inhibitor to offset the suppression of FLOT1 expression induced by siFLOT1. **b** and **c** Down-regulation of miR-608 could partially reverse the cell cycle arrest effect of siFLOT1 in UM-UC-3 cells. Error bars represent the S.D. from three independent experiments. *P < 0.05

To further confirm the function of FLOT1 in miR-608 induced growth inhibition, we transfected T24 and UM-UC-3 cells with siFLOT1, a small interfering RNA against FLOT1, to specifically knock down the expression of FLOT1. The transfection of siFLOT1 remarkably suppressed the proliferation and induced G1-phase arrest in BCa cells (Fig. 6a, b and c), as well as down-regulated the mRNA and protein levels of FLOT1 simultaneously (Fig. 6e), which was consistent with the effects of miR-608 on BCa cells. Moreover, knock-down of FLOT1 also activated the AKT/FOXO3a signaling by significantly down-regulating the levels of p-AKT and p-FOXO3a in BCa cells (Fig. 6d). Likewise, the expression levels of the downstream effectors of AKT/FOXO3a signaling, CCND1, CDK4, p-Rb and E2F1, which are involved in the regulation of cell proliferation and cell cycle progression, were also dramatically down-regulated in the FLOT1 silenced BCa cells (Fig. 6d, f and g). In addition,we co-transfected T24 and UM-UC-3 cells with another 3 different non-overlapping siFLOT1 together (Additional file 5: Table S2) to reproduce the suppression of FLOT1 and rule out off-target effects, which showed the consistent effects on the expression of FLOT1 and AKT/FOXO3a signaling pathway (Additional file 6: Figure S4), and also on the cell cycle progression (Additional file 7: Figure S5). At last, we co-transfected UM-UC-3 cells with miR-608 inhibitor. It showed that the down-regulation of miR-608 could offset the suppression of FLOT1 expression induced by siFLOT1 (Fig. 7a) and partially reverse the G1-phase arrest (Fig. 7b and c) and the p-FOXO3a down-regulation (Additional file 8: Figure S6) caused by siFLOT1. We also performed the co-transfection of pFLOT1 to abolish the expression inhibition of FLOT1 caused by miR-608. It appeared that overexpression of FLOT1 could reverse the suppression of cell proliferation caused by miR-608 (Additional file 9: Figure S7).

## Discussion

Lately, a growing number of studies revealed the potency of microRNAs as significant diagnostic or prognostic biomarkers and promising therapeutic targets in the treatment of malignant tumors. Meanwhile, the dysregulations of miRNAs in BCa have been extensively profiled as well, but the actual functions of most abnormally expressed miRNAs in the proliferation, progression and metastasis of BCa are still unclear. Human miR-608 is transcribed from 10q24.31 chromosomal locus which belongs to the non-coding region of SEMA4G gene. Although it has been reported that miR-608 is widely down-regulated and acts as a crucial tumor suppressor in multiple malignant tumors such as liver cancer, colon cancer, lung cancer and glioma [24–27], the expression pattern of miR-608 and its role in the tumorigenesis of BCa haven’t been verified yet.

In our study, we found that the basic expression levels of miR-608 in BCa tissues and BCa cell lines were drastically down-regulated as expected, compared with adjacent normal urothelial tissues and the normal urothelial cell line respectively. However, the mechanism of miR-608 down-regulation in various human tumors remains unknown. We are the first to report that the methylation status of CpG islands was involved in the epigenetic regulation of miR-608 expression in BCa cells.

Gain-of-function and loss-of-function studies of miR-608 were both conducted in BCa cell lines. The results revealed that miR-608 could suppress the proliferation and tumorigenesis of BCa cells in vitro and in vivo*,* which suggested miR-608 as a tumor suppressor in BCa. The mechanism of miR-608 induced inhibition of cell proliferation could at least partially be due to the G1 phase arrest caused by the activation of AKT/FOXO3a signaling pathway. Previous studies have proved that PI3K/AKT pathway played a key role in the regulation of G1 phase cell cycle progression [40]. As an important transcription factor, FOXO3a is a major downstream effector which is negatively regulated by PI3K/AKT signaling in various human cancers, and the phosphorylation of FOXO3a catalyzed by p-AKT will markedly suppress its (FOXO3a) transcriptional activity [36, 37, 41]. Inhibition of PI3K/AKT signaling pathway by down-regulating the level of p-AKT significantly activates FOXO3a which suppresses the expression of CCND1 and other related cell cycle regulators by inducing the up-regulation of tumor suppressing genes (p21 and p27) and finally inhibits the proliferation of cancer cells [33–35, 42]. In our study, we discovered that the overexpression of miR-608 could down-regulate the level of p-AKT and strongly enhance the transcriptional activity of FOXO3a in BCa cells, which revealed a new mechanism in the regulation of BCa cells proliferation.

Based on the basic principles of interactions between miRNA and mRNA and the effect of miR-608 on AKT/FOXO3a pathway, we then investigated the exact mechanism of miR-608 in regulating the proliferation of BCa cells. Finally, we identified flotillin-1 (FLOT1) as a key target of miR-608 responsible for its role in growth inhibition. FLOT1 was reported as a scaffolding protein of lipid raft microdomains and a highly conserved lipid raft maker, furthermore, it widely existed in cell membranes of different tissues and played important roles in signaling transduction, cell adhesion, cytoskeleton remodeling and endocytosis [43–47]. In addtion, FLOT1 was primarily known as a cell signaling mediator by anchoring various receptors of signaling pathways onto cell membrane [48, 49]. Previous studies showed that FLOT1 was constantly overexpressed in various cancers such as colorectal tumor, esophageal squamous carcinoma, tongue squamous carcinoma, prostate cancer, bladder transitional cell carcinoma, renal cell carcinoma and breast cancer [31, 38–40, 50–52]. Moreover, the overexpression of FLOT1 could dramatically promote the proliferation of prostate and bladder cancer cells, and also accelerate the invasion, migration of bladder cancer cells [38, 52]. The expression levels of FLOT1 in bladder and breast cancers were negatively correlated with the prognosis of patients [38, 39]. Further in vitro experiments proved that the down-regulation of FLOT1 in renal and breast cancers could inhibit the proliferation of cancer cells via activating AKT/FOXO3a signaling pathway [31, 39], which is consistent with the results of our study in bladder cancer cells. All these evidences suggested that FLOT1 acted as an oncogene in the tumorigenesis in many kinds of cancers, and might be a novel therapeutic target in the treatment of malignant tumors.

In our study, we also found the overexpression of FLOT1 in BCa tissues in contrast with paired adjacent non-tumor tissues, and the down-regulation of FLOT1 could sharply inhibit the proliferation of BCa cells via activating AKT/FOXO3a signaling pathway. Moreover, in BCa cells, we proved that the expression of FLOT1 was directly inhibited by miR-608, the down-regulation of FLOT1 and the G1 phase arrest induced by siFLOT1 could be significantly reversed by miR-608 inhibitor. Similarly, the suppression of cell proliferation caused by miR-608 could also be reversed by the overexpression of FLOT1. In conclusion, all the findings implied that miR-608 suppressed the tumorigenesis and proliferation of BCa cells in vitro and *vivo* by directly targeting the 3′-UTR of FLOT1 mRNA, and revealed a new downstream regulatory pathway of FLOT1 in BCa cells.

## Conclusions

Our study proved that miR-608 was a potential tumor suppressor in BCa. miR-608 could inhibit the tumorigenesis and proliferation of BCa cells by targeting the 3′-UTR of FLOT1. Despite the absence of further studies to identify other direct targets of miR-608, our experiments preliminarily indicated that the restoration of miR-608 might be a promising therapeutic option for BCa.

## Methods

### Cell lines and cell culture

Two human bladder cancer cell lines (T24 and UM-UC-3) and one non-tumor urothelial cell line (SV-HUC-1) were purchased from the Shanghai Institute of Cell Biology, Shanghai, China. All the cell lines were cultured in RPMI-1640 medium supplemented with 10% heat-inactivated fetal bovine serum in a humidified atmosphere with 5% CO2 at 37 °C.

### Clinical tissue samples

Paired BCa tissues and adjacent non-tumor bladder mucosal tissues were obtained from patients undergoing radical cystectomy. The samples were collected between January 2011 and June 2011 at the First Affiliated Hospital of Zhejiang University, after informed consent and Ethics Committee’s approval. The clinical data of the patients has been listed in Additional file 1: Table S1. Tissue samples were snap frozen in liquid nitrogen until RNA extraction.

### Chromogenic in Situ Hybridization (CISH) staining

Chromogenic in situ hybridization (CISH). A 5′-DIG and 3′-DIG-labeled, locked nucleic acid-incorporated miRNA probe (miRCURY LNATM Detection probe, Exiqon, Woburn, MA, USA) was used for the visualization of miR-608 in the bladder cancer TMAs which contained 46 cases with paired tumor and adjacent non-tumor tissues and 13 cases without corresponding non-tumor tissues were analyzed in this study. TMA was obtained from Xinchao Biotech, Shanghai, China. Paraffin tissue slides were deparaffinized and digested with proteinase K for 6.5 min (15 μg/ml). The slides were then prehybridized in a hybridization solution at 50 °C for 1 h. Tissues were hybridized for 2 days in the presence of 10 ng, 3′-5′ DIG-labeled miR-608 LNA probes at 4 °C (500 nM). Slides were washed stringently for 20 min at 50 °C, and an immunological reaction was conducted using anti-DIG-AP Fab fragments according to the manufacturer’s protocol. The miR-608 expression was detected by the BCIP/NBT substrates (Boster Biological Technology, Wuhan, China). The strength of positivity was semi-quantified by considering both the intensity and proportion of positive cells. The sequence of miR-608 probe used in CISH staining are listed in Table 1.

**Table 1:** The oligonucleotides used in this study

Name^a^	Sequence (5′- > 3′)^b^
miR-608 mimics (Sense)	AGGGGUGGUGUUGGGACAGCUCCGU
NC (Sense)	ACUACUGAGUGACAGUAGA
miR-608 Inhibitor	ACGAGCUGUCCCAACACCACCCCU
Inhibitor NC	CAGUACUUUUGUGUAGUACAA
miR-608 F	AGGGGTGGTGTTGGGACAGCTCCGT
miR-608 probe^c^	ACGGA GCTGT CCCAA CACCA CCCCT
U6 F	TGCGGGTGCTCGCTTCGGCAGC
CDK4 F	ATGGCTACCTCTCGATATGAGC
CDK4 R	CATTGGGGACTCTCACACTCT
CCND1 F	GCTGCGAAGTGGAAACCATC
CCND1 R	CCTCCTTCTGCACACATTTGAA
FLOT1 F	CCCATCTCAGTCACTGGCATT
FLOT1 R	CCGCCAACATCTCCTTGTTC
GAPDH F	AAGGTGAAGGTCGGAGTCA
GAPDH R	GGAAGATGGTGATGGGATTT
FLOT1 UTR Wt-1 F	CTGTCCATTGACAGTGAGGTC**CCACCCC**TCATCTCTCCTTGCCAAATAG
FLOT1 UTR Wt-1 R	TCGACTATTTGGCAAGGAGAGATGA**GGGGTGG**GACCTCACTGTCAATGGACAGAGCT
FLOT1 UTR Mut-1 F	CTGTCCATTGACAGTGAGGTCGGTGGGGTCATCTCTCCTTGCCAAATAG
FLOT1 UTR Mut-1 R	TCGACTATTTGGCAAGGAGAGATGACCCCACCGACCTCACTGTCAATGGACAGAGCT
FLOT1 UTR Wt-2 F	CAGCCTTCTGATGATCCCACT**CCACCCC**ACCTCAACTTATTTAACTTCG
FLOT1 UTR Wt-2 R	TCGACGAAGTTAAATAAGTTGAGGT**GGGGTGG**AGTGGGATCATCAGAAGGCTGAGCT
FLOT1 UTR Mut-2 F	CAGCCTTCTGATGATCCCACTGGTGGGGACCTCAACTTATTTAACTTCG
FLOT1 UTR Mut-2 R	TCGACGAAGTTAAATAAGTTGAGGTCCCCACCAGTGGGATCATCAGAAGGCTGAGCT

^a^F: forward primer, R: reverse primer

^b^Restriction sites are in bold, Mutated sites are underlined

^C^5′-DIG and 3′ -DIG labeled

### Immunohistochemistry (IHC) staining

Tissue sections of xenograft tumors in nude mice and the same bladder cancer TMAs used in CISH staining were analyzed in this study. TMA was obtained from Xinchao Biotech, Shanghai, China. All the paraffin tissue sections were dewaxed and rehydrated. Antigen retrieval was performed by heating the slides in sodium citrate buffer (10 mM, pH 6.0). After blocking with bovine serum albumin (Sango Biotech, Shanghai, China), the slides were incubated with anti-Ki-67 (Cell Signaling Technology, Beverly, MA, USA), or anti-FLOT1 (Epitomics, Burlingame, CA, USA) overnight at 4 °C. The slides were then incubated with a secondary antibody of goat anti-rabbit HRP conjugate (Cell Signaling Technology, Beverly, MA, USA) for 1 h at room temperature. A DAB solution was used for brown color development. The strength of positivity was semi-quantified by considering both the intensity and proportion of positive cells.

### Transient transfection of miRNA mimic, inhibitor and small interfering RNA

The miR-608 mimic (named as miR-608) and the negative control duplex (named as NC) which was non-homologous to all human gene sequences were used for transient gain of function study. The mir-608 inhibitor oligo (named as miR-608 inhibitor) and inhibitor negative control oligo (named as inhibitor NC) were used for transient loss of function study. A small interfering RNA duplex (siRNA) targeting human FLOT1 mRNA was used for RNAi study (named as siFLOT1). All the RNA duplexes and RNA oligos were synthesized by Gene Pharma (Shanghai, China). The Lipofectamine 2000 reagent (Invitrogen, USA) was used for transient transfection following the manufacturer’s instructions. The RNA duplexes and RNA oligos used in transfection are listed in Table 1.

### RNA isolation and quantitative real-time PCR

MircoRNAs were extracted from cultured cell lines with the RNAiso kit for small RNA (Takara, China) and reversely transcribed into cDNA with the One Step PrimeScript miRNA cDNA Synthesis Kit (Takara, China). Total RNA was isolated with TRIzol reagent (Takara, China) and reversely transcribed into cDNAs with the PrimeScript RT reagent Kit (Takara, China). The resulting cDNAs were quantified with SYBR Green reagent (Takara, China) by using the ABI 7500 fast real-time PCR System (Applied Biosystems, USA). The relative expression levels of miRNAs (miR-608) and mRNAs (FLOT1, CDK4 and CCND1) normalized by small nuclear RNA U6 and GAPDH mRNA respectively were calculated with the 2^-ΔΔCt^ method. All the qPCR primers were provided by Sango Biotech (Shanghai, China). All primers used are listed in Table 1.

### Cell growth and cell viability assay

Bladder cancer cells (T24 and UM-UC-3) were plated in 96-well plates at the density of 5000 cells per well. After an overnight cultivation, all the cells were incubated with RNA duplexes (NC, miR-608 or siFLOT1) or RNA oligos (inhibitor NC or miR

-608 inhibitor) for 48-72 h. The concentration of RNA duplexes ranged from 25 to 75 nM, and the concentration of RNA oligos was 100 nM. As soon as reached the time limit of incubation, the medium were removed and a Cell Counting solution (WST-8, Dojindo Laboratories, Japan) was added to each well and incubated at 37 °C for another 2 h. The absorbance of the solution was measured at 450 nm with MRX II absorbance reader (Dynex Technologies, USA).

### Colony formation assay

T24 and UM-UC-3 cells were harvested 24 h after transfected with RNA duplexes (50 nM of NC, miR-608 or siFLOT1) or RNA oligos (100 nM of inhibitor NC or mir-608 inhibitor). All the cells were resuspended in RPMI-1640 medium supplemented with 10% FBS and seeded in 6-well plates at a density of 400 cells per well. After 10 days of culture under standard conditions, the colonies on the plates were fixed with absolute methanol for 15 min and stained with 0.1% crystal violet for 20 min. The colonies with diameters greater than 2 mm were counted.

### Cell cycle analysis by flow cytometry

48 h after the transfection of RNA duplexes (50 nM of NC, miR-608 or siFLOT1) or RNA oligos (100 nM of inhibitor NC or miR-608 inhibitor), or after the co-transfection of RNA duplexes and RNA oligos (FLOT1 rescue experiment), bladder cancer cells were harvested and washed with PBS and fixed with 75% ethanol at -20 °C. After 24 h fixation, the cells were washed with PBS again and stained with propidium iodide using the cell cycle and apoptosis analysis kit (Beyotime, China) for 30 min. Cell cycle features were analyzed by BD LSRII Flow cytometry system with FACSDiva software (BD Bioscience, USA). The data were analyzed by ModFit LT 3.2 software (Verity Software House, USA).

### In vivo tumorigenicity assays

Animal studies were carried out according to institutional guidelines. Male BALB/c-nude mice (4 weeks old) were purchased from Shanghai Experimental Animal Center, Chinese Academy of Sciences, Shanghai, China. UM-UC-3 cells (1 × 10^6^ in 100 μl PBS) were subcutaneously injected into the right flank of each mouse. When tumors were first palpable, the mice were intratumorally injected with 30 μg of Lipofectamine 2000-encapsulated miR-608 or NC every 3 days for 18 days. Tumor size was measured every 3 days. Tumor growth was monitored by caliper measurements of the two perpendicular diameters every 3 days, and the volume of the tumor was calculated with the formula V = (width^2^ × length × 0.5).

### 5-aza-dc treatment and DNA methylation analysis

T24 and UM-UC-3 cells were first treated with 5 μM 5-aza-2′-deoxycytidine (5-aza-dc) (Sigma, St Louis, MO, USA) for 4 days. Bisulfite-sequencing PCR (BSP) was used to assess the methylation levels of the CpG islands located near the TSS of miR-608 in these two BCa cell lines, before or after the treatment of 5-aza-dc. The primers (forward) 5′- ATTTTATTTTTTAAGTTGGGTTAGG -3′ and (reverse) 5′- CTAACCTCAATCTCTACTACTACAACTC -3′ were used to amplify the DNA sequences of CpG islands in PCR procedure. The PCR products were separated by 3% agarose gel electrophoresis, extracted and then cloned into the pUC18 T-vector (Sangon, Shanghai, China). After bacterial amplification of the cloned PCR fragments by standard procedures, 10 clones were subjected to DNA sequencing (Sangon, Shanghai, China).

### Western blot analysis

All the cells were gathered and lysed in cell lysis buffer 48 h after the transfection. The BCA Protein Assay kit (Thermo Scientific, USA) was used to calculate the total protein concentration in every lysate. The same amounts of protein samples were separated by 10% SDS-polyacrylamide gels and transferred to polyvinylidene fluoride (PVDF) membranes. The membranes were blocked with 5% non-fat milk for 1 h, and incubated overnight with primary antibodies including anti-GAPDH, anti-FLOT1, anti-FOXO3a, anti-p-FOXO3a (Ser253), anti-AKT, anti-p-AKT (Ser473), anti-CCND1, anti-CDK4, anti-E2F1, anti-Rb, anti-p-Rb (Epitomics, Burlingame, CA). After being washed in TBS-T for three times, PVDF membranes were incubated with horseradish peroxidase (HRP)-conjugated goat anti-rabbit secondary antibody at a 1:5000 dilution for 1 h. The binding secondary antibody was detected by the enhanced chemiluminescence (ECL) system (Pierce Biotechnology, Rockford, USA).

### Dual-luciferase reporter assay

The 3′-UTR segments of FLOT1 including the wild type or the mutant type of miR-608 binding sites were cloned into the downstream of the luciferase reporter, the pmirGLO Dual-Luciferase miRNA Target Expression Vector (Promega, Madison, USA), between the SacI and SalI sites and verified by sequencing. HEK 293 T cells were plated into a 24-well plates and transfected with 50 nM miR-608 or NC and 100 ng of the luciferase vector (pmirGLO). Cells were harvested 48 h after the transfection. The relative luciferase activity was measured by the Dual-Glo luciferase assay kit (Promega).

### FLOT1 rescue experiment

miR-608 inhibitor or inhibitor NC were co-transfected with siFlot1 or NC in UM-UC-3 cells to evaluate whether the inhibition of miR-608 could offset the suppression of FLOT1 expression induced by siFLOT1. The FLOT1 overexpression plasmid (pFLOT1) was constructed by inserting the human FLOT1 complementary DNA lacking the 3′-UTR into the pIRES2-EGFP (Clontech, USA). miR-608 or NC was co-transfected with pFLOT1 or empty vector (pNull) in UM-UC-3 cells to assess whether overexpression of FLOT1 could reverse the suppression of cell proliferation caused by miR-608. The cells were harvested 48 h after the transfection and analyzed by subsequent Western blotting and cell cycle analysis.

### Statistical analysis

The experimental data were presented as the mean ± SD. Kolmogorov-Smirnov test was firstly used to determine the normality of the distribution of data in each group. Differences between two normal distribution groups were estimated using Student’s *t*-test. Differences among three or more normal distribution groups were analyzed using ANOVA test. Differences between abormal distribution groups were analyzed using non-parametric test (rank sum test, *χ* ^2^-test). All analyses were performed by using SPSS16.0 software (IBM, Armonk, NY, USA) and a two-tailed *P*-value < 0.05 was considered statistically significant.

## Additional files

Additional file 1: **Table S1.** Patients and tumor characteristics (*n* = 13). (DOCX 13 kb)

Additional file 2: **Figure S1.** The expression of miR-608 after the transfection of miR-608 mimic was quantified by qRT-PCR. Error bars represent the S.D. from three independent experiments. *P < 0.05. (JPG 307 kb)

Additional file 3: **Figure S2.** Western blot analysis showed that LY294002 significantly activated FOXO3a. (JPG 743 kb)

Additional file 4: **Figure S3.** The level of miR-608 after the transfection of miR-608 inhibitor was assessed by qRT-PCR. Error bars represent the S.D. from three independent experiments. *P < 0.05. (JPG 292 kb)

Additional file 5: **Table S2.** Sequences of 3 different non-overlapping siFLOT1. (DOCX 13 kb)

Additional file 6: **Figure S4.** Western blot analysis showed that the co-transfection of another 3 different non-overlapping siFLOT1 could also significantly down-regulate AKT/FOXO3a signaling related proteins in T24 and UM-UC-3 cell lines. (JPG 783 kb)

Additional file 7: **Figure S5.** Flow cytometric analysis showed that the co-transfection of another 3 different non-overlapping siFLOT1 together could also cause the similar G1 arrest as miR-608 did in Bca cells. Error bars represent the S.D. from three independent experiments. *P < 0.05. (JPG 138 kb)

Additional file 8: **Figure S6.** Western blot analysis showed that the down-regulation of miR-608 could partially reverse the p-FOXO3a down-regulation caused by siFLOT1. (JPG 218 kb)

Additional file 9: **Figure S7.** Flow cytometric analysis showed that the overexpression of FLOT1 by pFLOT1 could reverse the suppression of cell proliferation caused by miR-608. Error bars represent the S.D. from three independent experiments. *P < 0.05. (JPG 268 kb)

## Abbreviations

5-aza-dc: 5-aza-2′-deoxycytidine
BCa: Bladder cancer
BSP: Bisulfite sequencing PCR
CCK-8: Cell Counting Kit-8
CISH: Chromogenic in situ hybridization
IHC: Immunohistochemistry
miRNA: MicroRNA
qRT-PCR: Quantitative RT-PCR
TMA: Tissue microarray
TSS: Transcription start site
UTR: Untranslated region
