# Supplementary material for: MicroRNA-608 inhibits proliferation of bladder cancer via AKT/FOXO3a signaling pathway
Source: Mol Cancer. 2017 May 26;16:96. doi: 10.1186/s12943-017-0664-1 (PMC5446711; doi:10.1186/s12943-017-0664-1)
Supplement: Supplementary file 1 — Table S1. Patients and tumor characteristics (n = 13). (DOCX 13 kb) [file 12943_2017_664_MOESM1_ESM.docx]

**Table S1** Patients and tumor characteristics (n = 13)

| No. | Gender | Age | TNM Stage | Grade |
| --- | --- | --- | --- | --- |
| 1 | Male | 56 | T2N0M0 | Ⅲ |
| 2 | Male | 72 | T3N0M0 | Ⅲ |
| 3 | Female | 74 | T2N0M0 | Ⅲ |
| 4 | Female | 69 | T2N0M0 | Ⅱ |
| 5 | Male | 76 | T3N0M0 | Ⅲ |
| 6 | Male | 53 | T1N0M0 | Ⅲ |
| 7 | Male | 65 | T2N0M0 | Ⅱ |
| 8 | Male | 86 | T1N0M0 | Ⅲ |
| 9 | Male | 78 | T1N0M0 | Ⅱ |
| 10 | Male | 62 | T2N0M0 | Ⅲ |
| 11 | Male | 60 | T1N0M0 | Ⅰ |
| 12 | Female | 76 | T3N0M0 | Ⅲ |
| 13 | Male | 55 | T1N0M0 | Ⅱ |
