# Supplementary figures and images for: MicroRNA-608 inhibits proliferation of bladder cancer via AKT/FOXO3a signaling pathway
Source: Mol Cancer. 2017 May 26;16:96. doi: 10.1186/s12943-017-0664-1 (PMC5446711; doi:10.1186/s12943-017-0664-1)

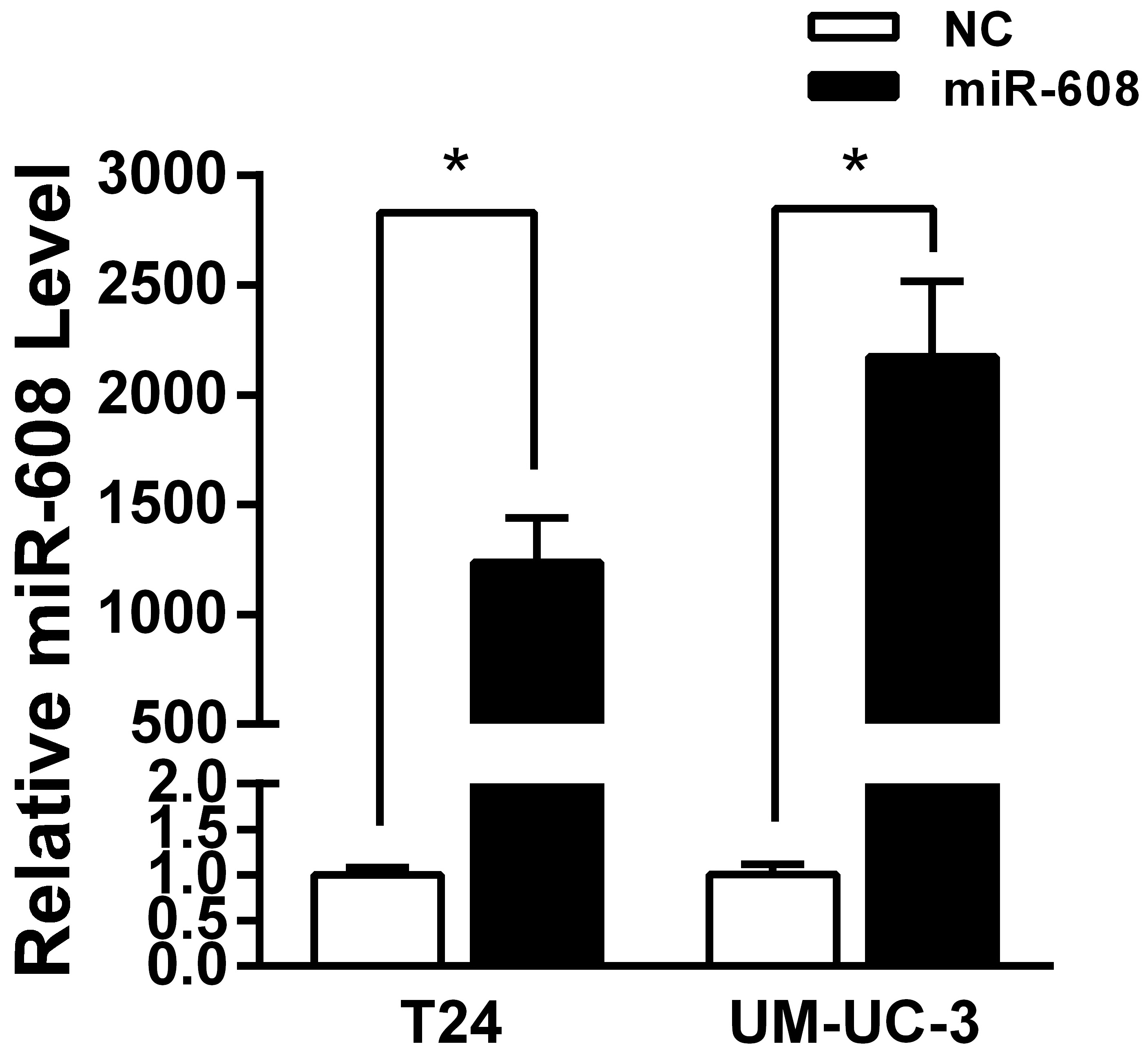

Supplement: Supplementary file 2 — Figure S1. The expression of miR-608 after the transfection of miR-608 mimic was quantified by qRT-PCR. Error bars represent the S.D. from three independent experiments. *P < 0.05. (JPG 307 kb) [file 12943_2017_664_MOESM2_ESM.jpg]

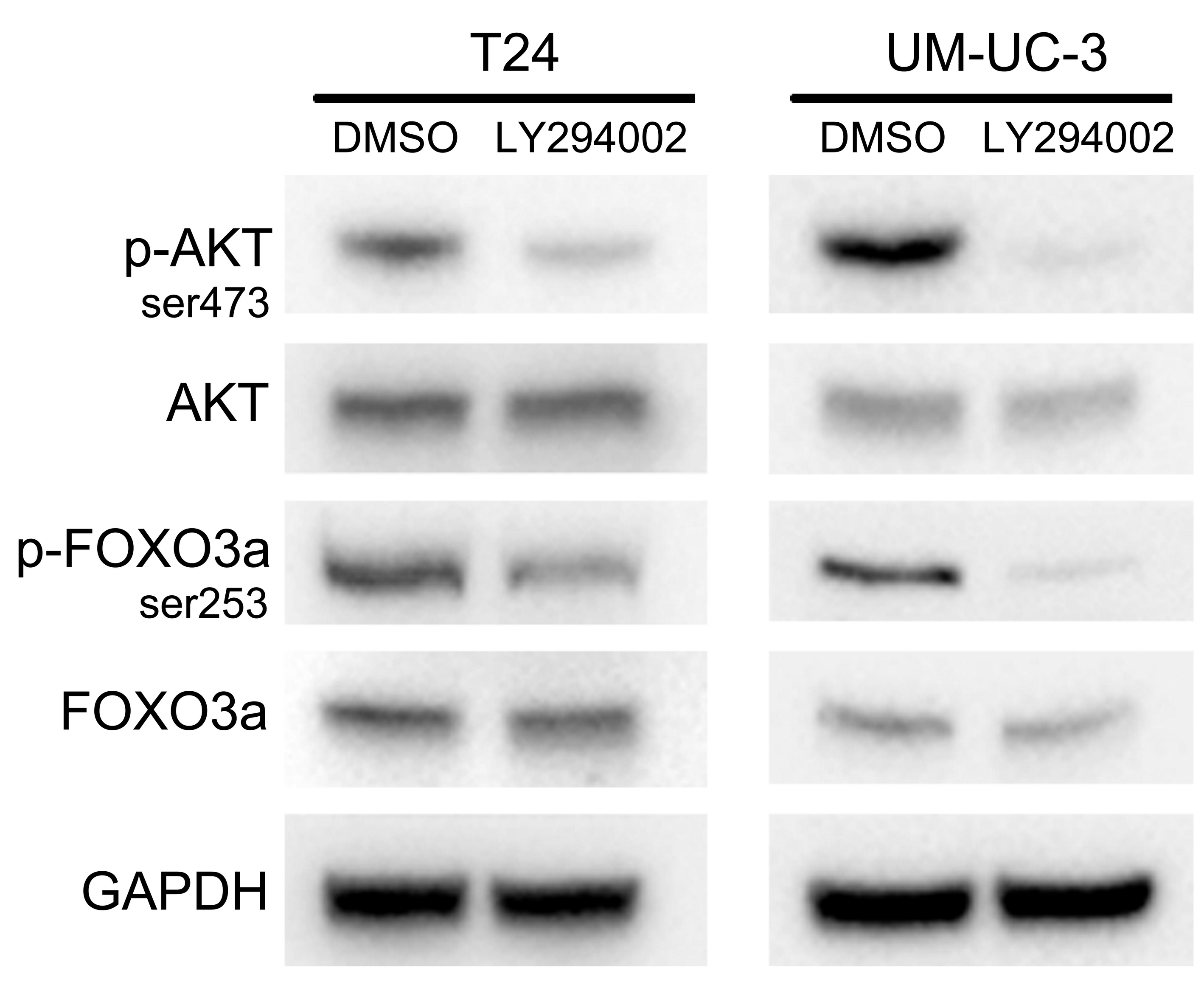

Supplement: Supplementary file 3 — Figure S2. Western blot analysis showed that LY294002 significantly activated FOXO3a. (JPG 743 kb) [file 12943_2017_664_MOESM3_ESM.jpg]

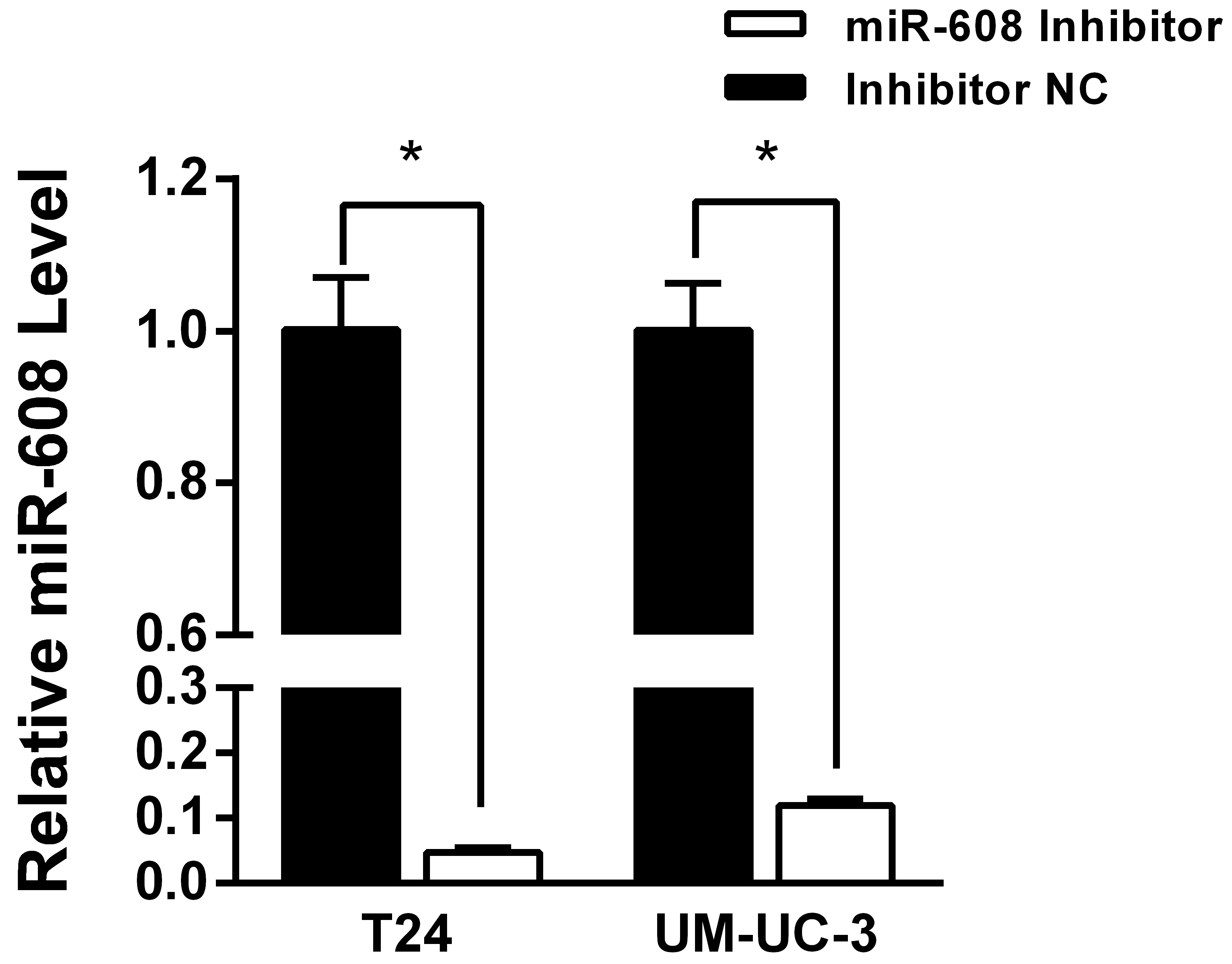

Supplement: Supplementary file 4 — Figure S3. The level of miR-608 after the transfection of miR-608 inhibitor was assessed by qRT-PCR. Error bars represent the S.D. from three independent experiments. *P < 0.05. (JPG 292 kb) [file 12943_2017_664_MOESM4_ESM.jpg]

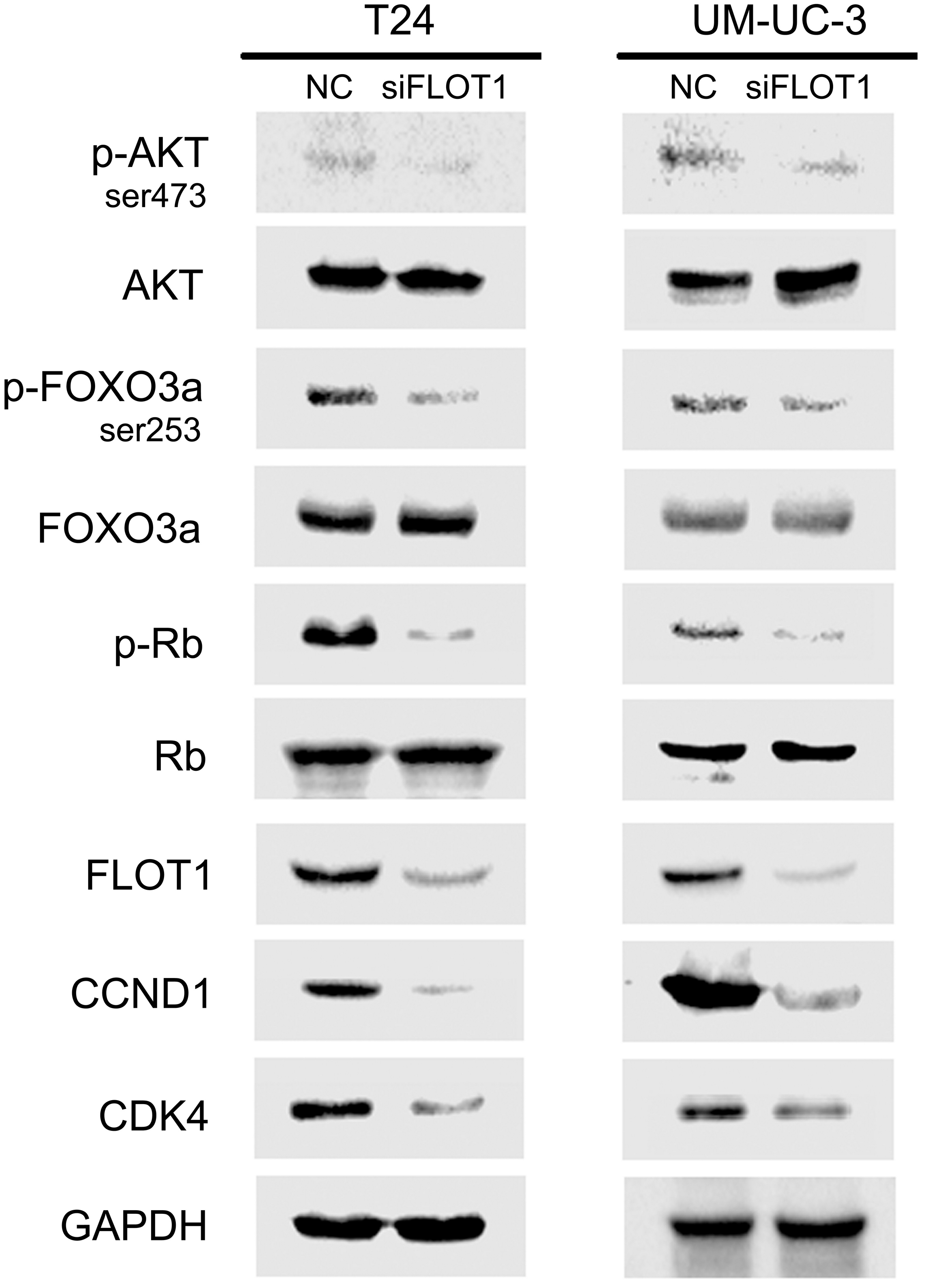

Supplement: Supplementary file 6 — Figure S4. Western blot analysis showed that the co-transfection of another 3 different non-overlapping siFLOT1 could also significantly down-regulate AKT/FOXO3a signaling related proteins in T24 and UM-UC-3 cell lines. (JPG 783 kb) [file 12943_2017_664_MOESM6_ESM.jpg]

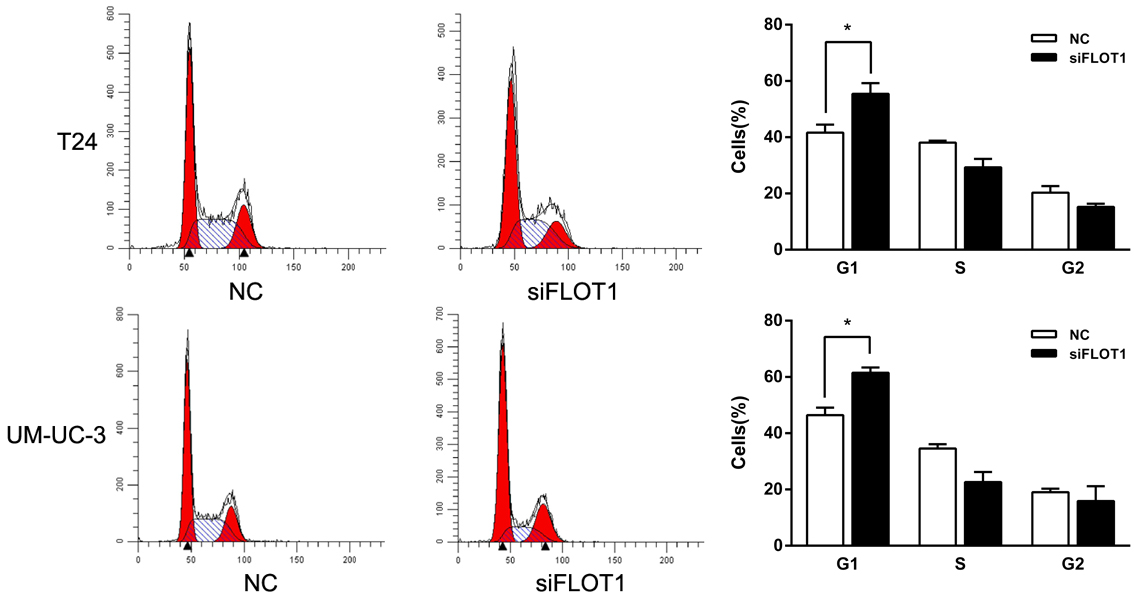

Supplement: Supplementary file 7 — Figure S5. Flow cytometric analysis showed that the co-transfection of another 3 different non-overlapping siFLOT1 together could also cause the similar G1 arrest as miR-608 did in Bca cells. Error bars represent the S.D. from three independent experiments. *P < 0.05. (JPG 138 kb) [file 12943_2017_664_MOESM7_ESM.jpg]

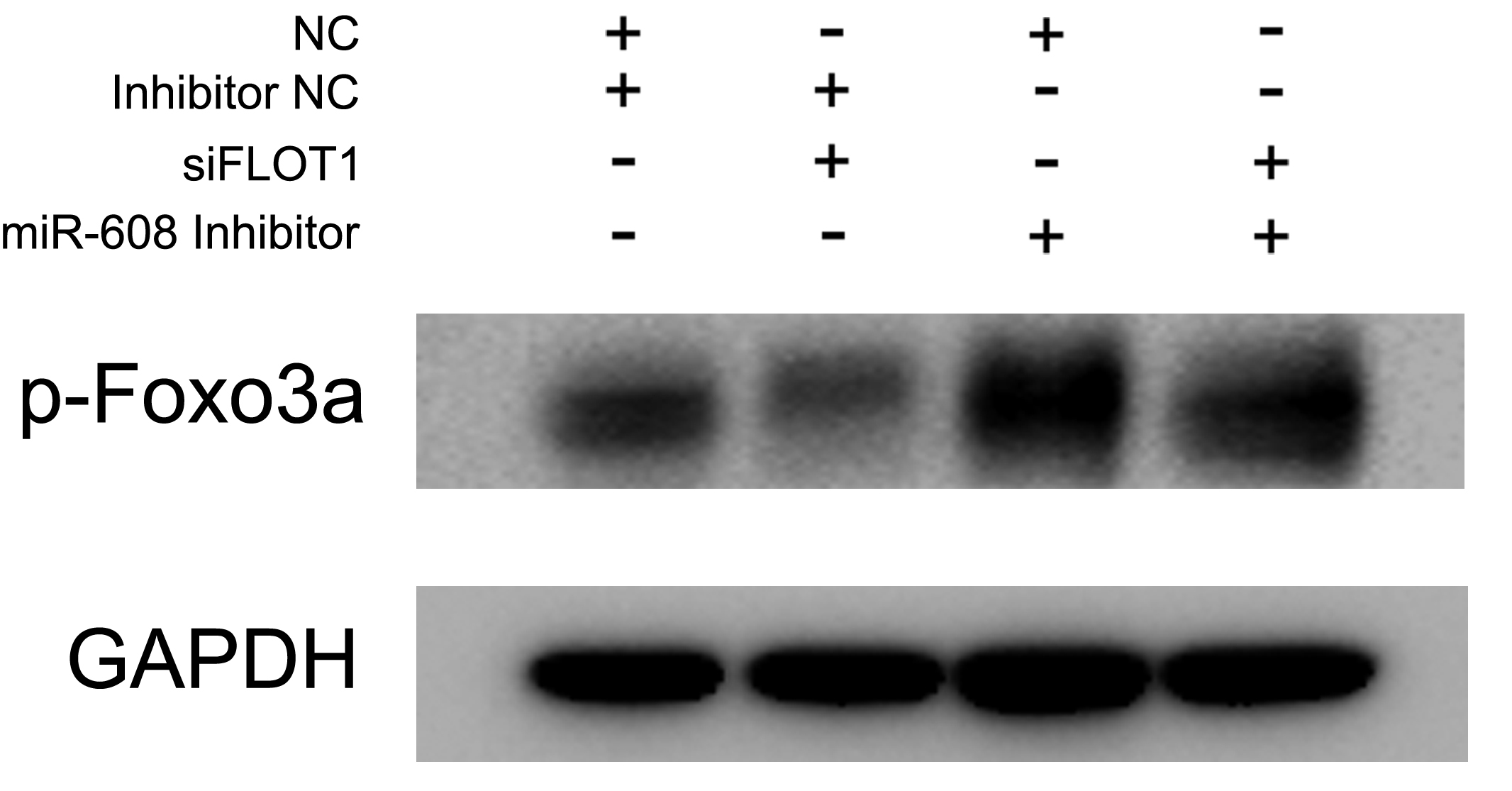

Supplement: Supplementary file 8 — Figure S6. Western blot analysis showed that the down-regulation of miR-608 could partially reverse the p-FOXO3a down-regulation caused by siFLOT1. (JPG 218 kb) [file 12943_2017_664_MOESM8_ESM.jpg]

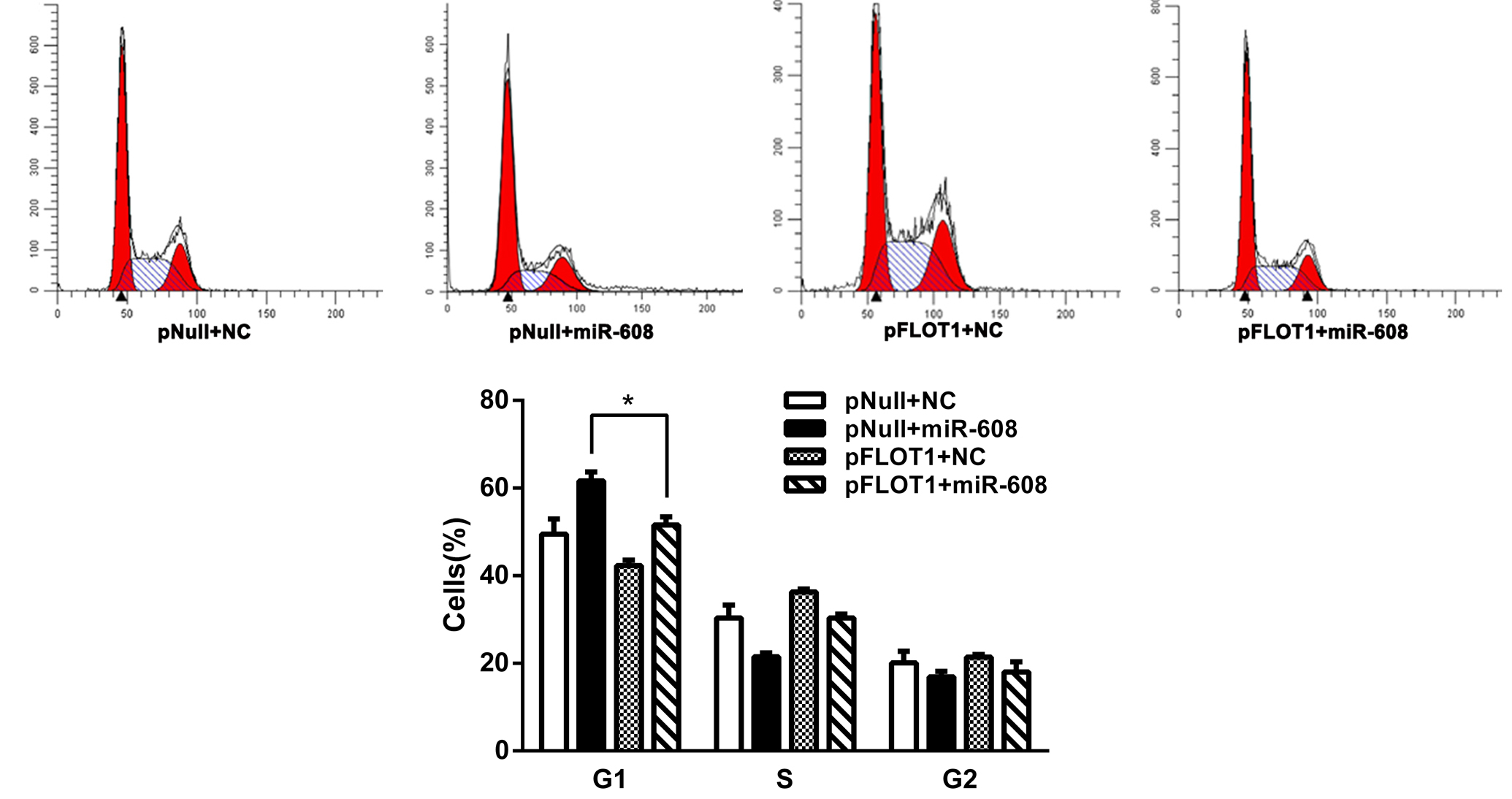

Supplement: Supplementary file 9 — Figure S7. Flow cytometric analysis showed that the overexpression of FLOT1 by pFLOT1 could reverse the suppression of cell proliferation caused by miR-608. Error bars represent the S.D. from three independent experiments. *P < 0.05. (JPG 268 kb) [file 12943_2017_664_MOESM9_ESM.jpg]
