# Supplementary material for: MicroRNA-608 inhibits proliferation of bladder cancer via AKT/FOXO3a signaling pathway
Source: Mol Cancer. 2017 May 26;16:96. doi: 10.1186/s12943-017-0664-1 (PMC5446711; doi:10.1186/s12943-017-0664-1)
Supplement: Supplementary file 5 — Table S2. Sequences of 3 different non-overlapping siFLOT1. (DOCX 13 kb) [file 12943_2017_664_MOESM5_ESM.docx]

**Table S2** Sequences of 3 different non-overlapping siFLOT1

| No. | Sense（5'-3'） | Antisense（5'-3'） |
| --- | --- | --- |
| 1 | GGCAGAAAUUCUCAGAACATT | UGUUCUGAGAAUUUCUGCCTT |
| 2 | GCUACACUCUGAAGGACAUTT | AUGUCCUUCAGAGUGUAGCTT |
| 3 | GAGAUUACGAACUGAAGAATT | UUCUUCAGUUCGUAAUCUCTT |
